# Shear induced carboplatin binding within the cavity of a phospholipid mimic for increased anticancer efficacy

**DOI:** 10.1038/srep10414

**Published:** 2015-05-22

**Authors:** Jingxin Mo, Paul K. Eggers, Xianjue Chen, Muhammad Rizwan Hussain Ahamed, Thomas Becker, Lee Yong Lim, Colin L. Raston

**Affiliations:** 1 Pharmacy, School of Medicine and Pharmacology, The University of Western Australia, 35 Stirling Highway, Crawley, WA, 6009, Australia; 2 Centre for NanoScale Science and Technology, School of Chemical and Physical Sciences, Flinders University, Bedford Park, SA, 5042, Australia; 3 School of Chemistry and Biochemistry, The University of Western Australia, 35 Stirling Highway, Crawley, WA, 6009, Australia; 4 Nanochemistry Research Institute, Department of Chemistry, Curtin University, Perth, WA 6845, Australia

## Abstract

Vesicles 107 ± 19 nm in diameter, based on the self-assembly of tetra-*para*-phosphonomethyl calix[4]- arene bearing n-hexyl moieties attached to the phenolic oxygen centres, are effective in binding carboplatin within the cavity of the macrocycle under shear induced within a dynamic thin film in a continuous flow vortex fluidic device. Post shearing the vesicles maintain similar diameters and retain carboplatin within the cavity of the calixarene in a hierarchical structure, with their size and morphology investigated using DLS, TEM, SEM and AFM. Location of the carboplatin was confirmed using NMR, FTIR, ESI-MS and EFTEM, with molecular modelling favouring the polar groups of carboplatin hydrogen bonded to phosphonic acid moieties and the four member cyclobutane ring directed into the cavity of the calixarene. The loading efficiency and release profile of carboplatin was investigated using LC-TOF/MS, with the high loading of the drug achieved under shear and preferential released at pH 5.5, offering scope for anti-cancer drug delivery. The hierarchical structured vesicles increase the efficacy of carboplatin by 4.5 fold on ovarian cancer cells, lowered the IC_50_ concentration by 10 fold, and markedly increased the percent of cells in the S-phase (DNA replication) of the cell cycle.

Cancer is one of the biggest killers worldwide with about 8 million people dying from cancer every year^1^. Platinum based drugs have received worldwide approval in treating cancers, with carboplatin as a second-generation platinum based drug being safer than the traditional cisplatin while retaining a similar spectrum of activity. The anti-cancer activity of carboplatin arises from its binding to DNA, thereby inhibiting DNA synthesis and cell division^2^. However, there are two major limitations of carboplatin, its high polarity that hinders its permeation across cell membrane, and its facile degradation into non-active complexes by glutathione (γ-glutamylcysteinylglycine) and proteins containing methionine or cysteine residues. Significant breakthroughs in the next decade for platinum-based anti-cancer drugs are likely to come from improved chemical delivery of the already approved drugs. One delivery method is through vesicles^3^ made up of designer calix[4]arene based lipid mimics^3^ which are non-toxic^4^ and are potent anti-oxidants^5^. These vesicles can be wrapped with polymers for increased stability^3^ tagged with fluorescent molecules for tracking, tagged with targeting agents for increased delivery specificity^3^ and can intercalate non-polar drugs^5^ as well as store water-soluble active agents in their central core. Here we report on the ability of ca 100 nm vesicles to bind a carboplatin molecule within the ‘cup’ or cavity of the calix[4]arene lipid mimic, which is dramatically enhanced within the shear regime of the dynamic thin films in a vortex fluidic device^6^. Such vesicles offer scope for imparting added cancer cell specificity^7^ to a cancer targeting tag, as well as imparting a degree of protection to the carboplatin molecule during transport to the tumor. Indeed, as shown herein, the calix[4]arene lipid mimic incorporating carboplatin significantly increases the anti-cancer efficacy of the drug.

## Results and Discussion

### Design and synthesis of the calixarene

The calixarene lipid mimic (P4C6) featured herein is necessarily amphiphilic, with ionisable methylphosphonic acid groups (head groups) attached to the upper rim of a calix[4]arene scaffold and hydrophobic *n*-hexyl groups (tails) attached to the lower rim phenolic oxygen centers which also renders the calix[4]arene sterically restricted to the cone conformation. Such a calixarene can assemble in water to form micelles as small assemblies and/or vesicle bilayers as much larger assemblies^3^. For the latter, a close packed vesicle bilayer has the “cup” of the calixarene directed towards aqueous medium, for binding small drug molecules as host-guest complexes. P4C6 was prepared *via* modifying a method we have previously described for the synthesis of the corresponding *n*-dodecyl substituted calixarenes^3^ and *n*-octyl substituted calixarenes^5^. As described in detail in the ESI, this included standard calix[4]arene synthesis followed by addition of the *n-*hexyl groups through bromohexane and sodium hydride in DMF, formulation *via* the Duff reaction, reduction to the alcohol with sodium borohydride, chlorination with thionyl chloride, phosphorylation with triethylphosphite and deprotection to the final compound using bromotrimethylsilane.

### Vesicle formation and carboplatin loading

The vesicles were formed by the thin-film method described in the methods section. Dynamic light scattering (DLS) gave a hydrodynamic diameter of 107 ± 19 nm (Figure S1). The vesicles were loaded with carboplatin using a recently developed vortex fluidic device (VFD)^6^ which has a rapidly rotating tube open at one end (Figure S2). The high rotational speed generates shear in the resulting thin films arising from viscous drag, Stewartson/Ekman layers^6^, and resonance effect^8^, in tracking towards conditions beyond diffusion control. The constant shear in this microfluidic platform is effective in disassembling self-organized molecular capsules for small molecule inclusion or encapsulation,^9^,^10^,^11^,^12^ as has been established for the related spinning disc processor^13^. Moreover, the shear in the VFD is effective in being able to correctly refold proteins^14^. Controlling the re-organisation processes in the present study to bind the carboplatin was carried out in the VFD under the continuous flow mode. This mode incorporates scalability at the inception of the science and thus facilitates translation of the processing to industry requirements. In the continuous flow mode, jet feeds deliver reagents to the bottom of a rapidly rotating tube, typically borosilicate glass, as a standard 10 mm diameter NMR tube. Whirling of the liquid up the tube which is inclined relative to the horizontal position, creates the shear associated with the viscous drag within the dynamic thin film, and the liquid is collected at an exit outlet at the top. Specifically, in the present study, carboplatin dissolved in water (10 mM CPt) was delivered in one jet feed, and a solution of the sized vesicles (10 mM P4C6) discussed above was delivered in another and the combined solution was passed through the VFD three times. The rotational speed was set at 8000 rpm, the flow rate at 0.6 mL/min, and the inclination angle of the tube at 45°, which are operating parameters within the regime for other inclusion/encapsulation experiments^15^. As a control, the same amount of blank P4C6 vesicle and carboplatin were sonicated for 30 minutes (Elma Hans Schmidbauer GmbH & Co., Singen, Germany) at 80% magnitude, without shear in the VFD. This resulted in only 17% host-guest complex formation after 30 minutes, and with just batch stirring there is almost no complexation after 48 hours. The particle size for sonication is 119 ± 13 nm, is similar to that for the vesicles post VFD processing.

**Figure 1:**
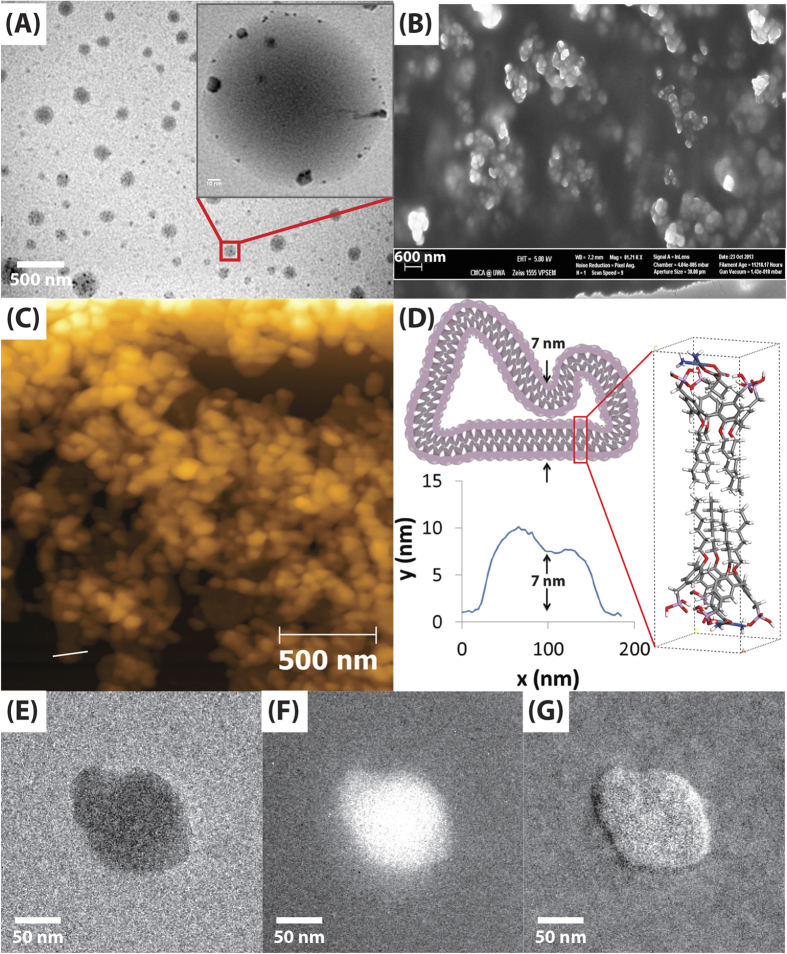
VFD processed P4C6-carboplatin host-guest vesicles: (**A**) TEM image, scale bar 500 nm (10 nm for inset), (**B**) SEM image, scale bar 600 nm, (**C**) AFM image, (**D**) sectional height profile of a collapsed vesicle and elemental mapping of host-guest complex with energy-filtered transmission electron microscopy (EFTEM) for (**E**) unfiltered, (**F**) carbon and (**G**) platinum.

DLS on the VFD processed P4C6 vesicles loaded with carboplatin gave a mean hydrodynamic diameter of 134 ± 25 nm (Figure S3), which is consistent with transmission electron microscopy (TEM), scanning electron microscopy (SEM) and atomic force microscopy (AFM), Fig. 1A-C respectively. From the AFM height profile, Fig. 1D, it appears that the vesicles are unilamellar, although multi-lamellar vesicles are also probable. As described in the ESI, micelles formed from the P4C6 *via* a base-acid technique are approximately 3 nm in diameter (Figure S4) which translates to an approximate bilayer thickness expected for the corresponding vesicles. The diameter of a collapsed vesicle shown in Fig. 1D is approximately 7 nm which is consistent with vesicles formed using the above thin film process having two bilayers or unilamellar prior to collapsing.

The presence of a 1:1 P4C6-carboplatin complex within the vesicles was demonstrated by electrospray ionization mass spectrometry (ESI-MS) with a peak at [1508.72 m/z] (Figure S5) which corresponds to the [P4C6+carboplatin+H]^+^ ion (calculated [1508.52 m/z]). There was no evidence for a 2:1 complex, which could result from the formation of a molecular capsule based on two calixarenes encapsulating a single molecule of carboplatin, as established for the next largest ring system, *p*-phosphonated calix^5^arene, in aqueous medium for the same drug molecule^16^. Energy filtered transmission electron microscopy (EFTEM) was used to demonstrate that carboplatin was evenly distributed across the vesicle, with the unfiltered TEM, the carbon map, and the platinum map of a vesicle displayed in Fig. 1E-G respectively. Elemental mapping of blank P4C6 vesicles with energy-filtered transmission electron microscopy (EFTEM) is presented in Figure S6.

**Figure 2:**
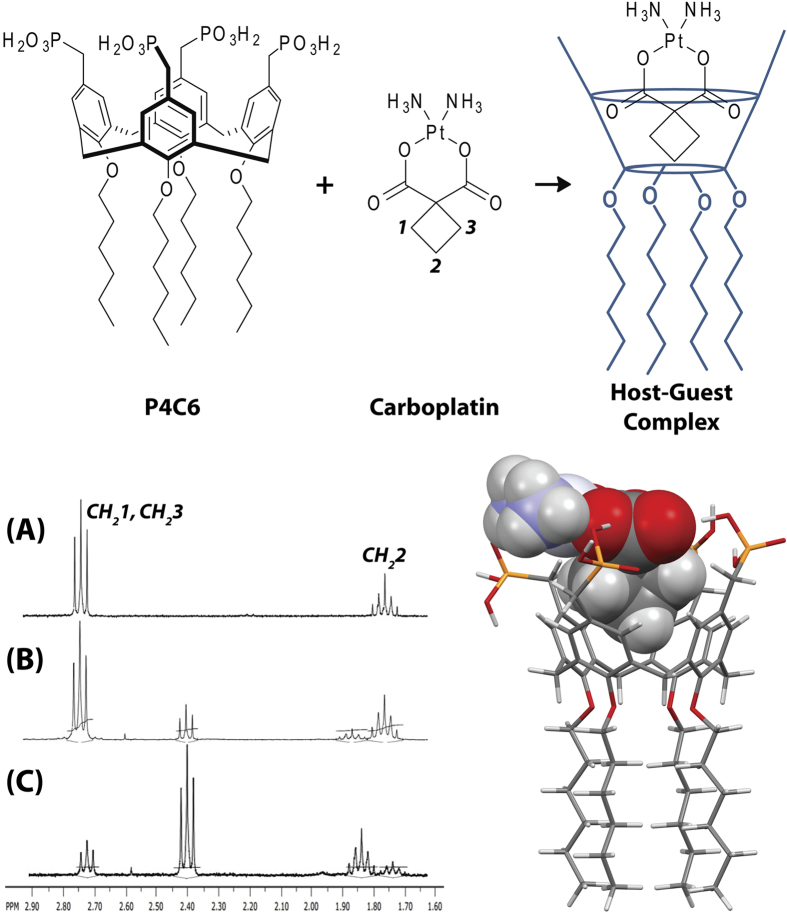
Schematic of the binding of carboplatin within the cavity of P4C6, MOPAC2012 PM7 geometry optimized model of the P4C6-carboplatin complex and ^1^H NMR spectra of (**A**) carboplatin, (**B**) carboplatin with 1.0 equivalent of P4C6 after probe sonication, and (**C**) carboplatin with 1.0 equivalent of P4C6 after VFD processing.

Fourier transform infrared spectroscopy (FTIR) shows peak shifts arising from the P4C6-carboplatin host-guest complex, in particular, a shift arising from NHOP hydrogen bond formation, with ν_a_(NH_3_) at 3253 s cm^−1^ (Figure S7A) shifted to 3339 s cm^−1^ (Figure S7C) on host-guest complex formation.^1^H NMR combined with FTIR can be used to gain insight into how the carboplatin sits in the cavity of the P4C6. The ^1^H NMR spectra of carboplatin alone and the P4C6 vesicles with carboplatin are shown in Fig. 2A-C.

Figure 2A shows the chemical shifts for the protons of carboplatin attached to C1, C2 and C3, with the triplet at 2.72 ppm assigned to the CH_2_ groups at C1 and C3 and the quintuplet at 1.74 ppm to the CH_2_ at C2. After host-guest complex formation (Fig. 2B,C) the carboplatin CH_2_ resonances for C1 and C3 shift 0.36 ppm upfield, while the CH_2_ resonance for C2 shifts 0.09 ppm downfield. The upfield shift is consistent with a shielding effect within the cavity of the P4C6, with the protons directed over the face of the aromatic rings. The deshielding of the C2 protons is consistent with the unique methylene group being directed to the centre of the cavity, closer to the O-alkyl chains. Combined with the shifts from hydrogen bonding for FTIR data, the most likely host-guest complex structure is shown in Fig. 2, along with an energy minimized molecular model.

The molecular model in Fig. 2 was produced by first minimizing the energy of free P4C6 and carboplatin individually using MOPAC2012 semi-empirical PM7. The minimized P4C6 and carboplatin were then manually docked together in the host-guest model, for binding and energy minimization. The optimized geometry gave a final heat of formation of -1168 Kcal/mol. The MOPAC2012 semi-empirical PM7 model of the host-guest complex indicates that the hydrophobic interaction between the carboplatin bridging ligand and the cavity of the P4C6 are complemented by six intermolecular hydrogen bonds between the nitrogen and oxygen atoms in carboplatin and the opposing phosphonic acid groups on the calixarene (NH···OP; 1.9-2.0 Å. POH···O; 1.7-2.1 Å). Indeed, the flexible phosphonate groups serve to anchor the drug molecule in the cavity through extended hydrogen bonding. As indicated by the molecular model, Fig. 2, the carboplatin C2 sits centrally within the expected deshielding region with the C1 and C3 protons located above the plane of calixarene phenolic groups, within the shielding region of the aromatic rings. This geometry is consistent with the above ^1^H NMR data, which shows a more pronounced upfield shift for the hydrogen atoms at C1 and C3, and a small downfield shift for the hydrogen atoms at C2, as well as the FTIR which shows a shift in ν(NH) arising from hydrogen bonding.

Integration of the ^1^H NMR spectrum established that the shear within the VFD gave 75% incorporation of the carboplatin within the cavity of the available P4C6, whereas probe sonication for 30 minutes resulted in only 17% incorporation (Fig. 2B,C). This is despite the short processing time for a finite volume of liquid moving through the VFD under continuous flow three times, being less than three minutes^6^. Presumably the intense micro-mixing and subsequent shear in the VFD results in the majority of the P4C6 molecules on the solution face of the bilayer (immediately accessible) to bind carboplatin. Given that 50% of the P4C6 cavities point towards the centre of the vesicle, and yet 75% of them become occupied with carboplatin, there must be some disruption of the bilayer taking place in the dynamic thin film produced by the VFD. Disassembly of the vesicles under shear can be ruled out since the size distribution of the vesicles remained relatively similar before and after VFD processing (Figure S1 and S3), and disassembly of the vesicle is expected to result in a large size distribution. The complex shear regimes produced by the VFD^6^ may induce an increase in lipid mobility where P4C6 molecules with the head groups facing the interior of the vesicles undergo a flip to the side facing the exterior and *vice versa*. This would allow P4C6-carboplatin host-guest complexes to be on the inside layer of the vesicle as well as the outside layer, in tracking towards 75% binding of the carboplatin molecules. The constant shear during the VFD processing allows for intimate contact of free carboplatin with the flipped calixarene molecules. In contrast the application of sonication induces localized short-lived cavitation events which are ineffective in exposing the inner calixarene cavities to the carboplatin.

The carboplatin loading in the P4C6 vesicles was also quantified by LC-TOF/MS using a literature adapted method^17^. Under set electrospray ionization conditions, carboplatin was predominantly the protonated fragment ([C_5_H_10_NO_2_Pt]^+^), and the MS parameters were optimized to maximize the response for the production of *m*/*z* 310.0152 (Figure S8). The standard curve for carboplatin was calculated to be y = 0.23x + 78.06 (R^2^ = 0.996) (Figure S9). The percent drug loading efficiency (*D*_*L*_) was calculated from the mass of carboplatin in a host-guest complex (*m*_*CPt*_) and the mass of host-guest complex (m_HG_) according to:
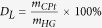


The loading efficiency of carboplatin within the cavity of the P4C6-carboplatin host-guest vesicles was established at 24 ± 2% (W/W), as determined for three separately prepared vesicles suspensions. This loading, which is higher than that determined using.^1^H NMR spectroscopy, equates to approximately 1.02 P4C6 per carboplatin molecule post shearing. We hypothesize that 75% of the associated carboplatin is encapsulated within a host-guest complex and the remaining 25% of the carboplatin is hydrogen-bonded to the surface without binding in the cavity of the calixarenes. Furthermore, the high rate of lipid flipping within the VFD processing may enable adsorbed carboplatin to be transferred to the centre of the vesicle.

### Release of carboplatin from P4C6-carboplatin host-guest vesicles

**Figure 3:**
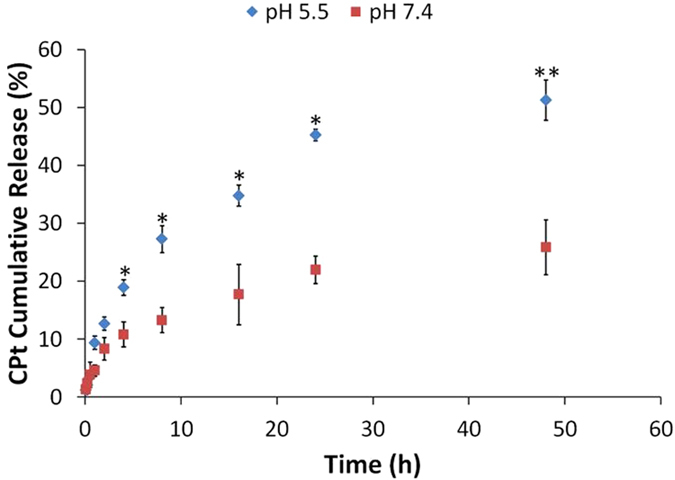
Release profile of carboplatin (CPt) from CPt-P4C6 host-guest vesicles at pH 7.4 and pH 5.5 over 48 h (*n *= 3) (Mean ± SD). Statistical analysis was performed using a Student’s t-test. Values of *P < 0.05 and **P < 0.01 were considered statistically significant.

In ascertaining the potential of the system for carboplatin delivery, its release profile from the host-guest vesicles was investigated using dialysis bags and PBS at pH 5.5 or 7.4 as receiving medium (Fig. 3).

The P4C6-carboplatin host-guest vesicles (CPt-P4C6) were relatively stable at pH 7.4, with about 20% of loaded carboplatin released after 2 days at 37 °C (Fig. 3). In contrast, at pH 5.5, approximately 20% of carboplatin was released from the vesicle during the initial 5 h, which represents a 10-fold faster release rate. The increased carboplatin release by the vesicles under acidic conditions relates to the pK_a_ of the phosphonic acid head groups which is 7.2^16^. Thus, at pH 7.4 the phosphonic acid head groups are deprotonated and hence take on a negative charge whereas at pH 5.5 the head groups are predominantly fully protonated and hence neutral. The loss of charge in acidic conditions is associated with a decrease in polarity of the head groups and suggests that the vesicles will be destabilized within an aqueous environment relative to the mildly basic pH 7.4. These findings are promising for targeting cancer chemotherapy as the pH within a tumor is typically between pH 5.5 and 7.0, compared to a pH of 7.5 in normal tissues^18^. This suggests that the vesicles will have a greater stability in systemic circulation at the pH of normal tissues, with potential for more rapid carboplatin release following extravasation to tumorigenic tissues where the pH decreases in the extracellular milieu and in cytoplasmic lysosome.

### Efficacy of P4C6-carboplatin host-guest vesicles

The efficacy of the P4C6-carboplatin vesicles was investigated through cell vitality studies on the SKOV-3 ovarian cancer cell line to calculate the half-maximal inhibitory concentration (IC_50_). The IC_50_ in this case gives a quantitative measure of the concentration of P4C6, free carboplatin (CPt), and carboplatin loaded in P4C6 vesicles (CPt-P4C6) required to kill 50% of the ovarian cancer cells. In preliminary experiments we found no significant difference (P > 0.05) between flow cytometry and MTT data. The IC_50_ of CPt, P4C6 and CPt-P4C6 against the SKOV-3 cells were determined using a FITC-Annexin V/Dead Cell Apoptosis Kit.

**Figure 4:**
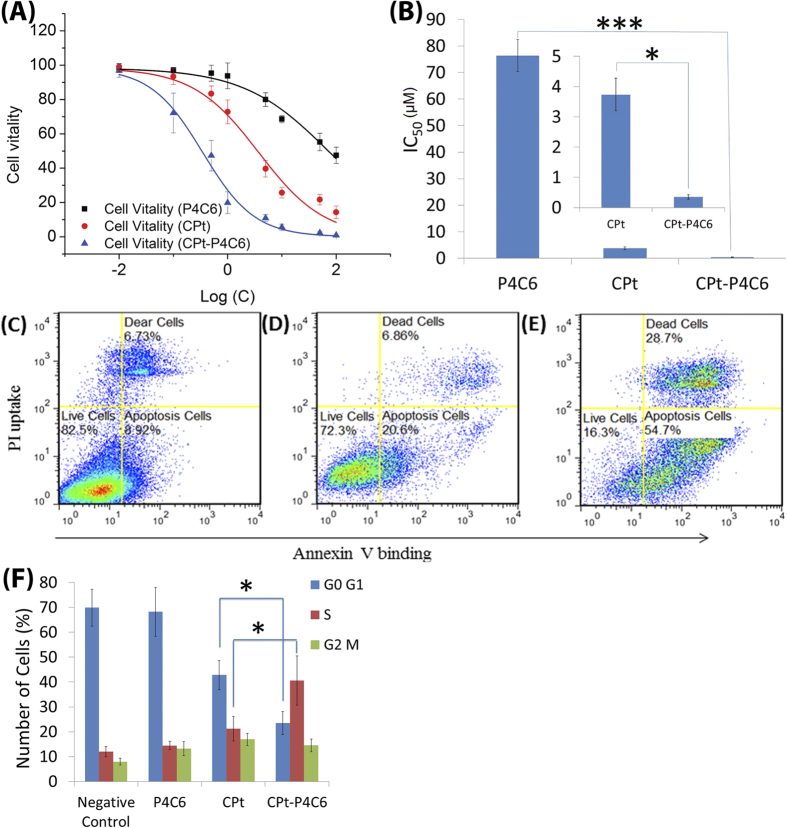
Carboplatin-P4C6 induced apoptosis of SKOV-3 cells and caused cell arrest in the S phase. (**A**) Dose-response curves for P4C6, CPt (=carboplatin) and CPt-P4C6 against SKOV-3 cells after 24 h incubation, and (**B**) corresponding IC_50_ for P4C6, CPt and CPt-P4C6. Flow cytometric analysis of SKOV-3 cells treated with (**C**) 5 μM P4C6, (D) 1 μM **C**Pt, and (**E**) 1 μM CPt-P4C6 for 24 h followed by treatment with the reagents in the Dead Cell Apoptosis Kit. (**F**) Cell cycle analysis of SKOV-3 cells treated for 12 h with negative control (cell culture media), 5 μM P4C6, 1 μM CPt, and 1 μM CPt-P4C6. Statistical analysis was performed using a Student’s t-test. Values of *P < 0.05 and ***P < 0.005 were considered statistically significant.

As can be seen in Fig. 4A-E, the IC_50_ data in Fig. 4B demonstrates that for the same cytotoxic effect a 10 fold lower concentration of carboplatin is required if it is combined with the P4C6 vesicle (3.8 μM for CPt vs. 0.35 μM for CPt-P4C6). Furthermore, this is not due to toxicity of the P4C6 as a 200 fold higher concentration of P4C6 alone (78 μM) is required to get the same effect as CPt-P4C6. This high efficacy is also reflected in the flow cytometric analysis shown in Fig. 4C-E where CPt-P4C6 results in 16.3% live cells at 1 μM, compared to 72.3% live cells at 1 μM of CPt. Thus there is a 4.5 fold increase in cell mortality by loading the carboplatin into the vesicles. This increase is also not due to the toxicity of P4C6, with the flow cytometric analysis results showing 82.5% live cells for P4C6 at 5 μM. The collective data suggests that CPt is more efficiently delivered into the cells when it is associated with P4C6.

### Mechanism of action for the P4C6-carboplatin host-guest vesicles

CPt binds with DNA to form intrastrand crosslinks and adducts which activates a number of biochemical pathways that lead eventually to cell cycle arrest in the S phase, followed by cell recovery, apoptosis and cell death depending on the extent of DNA damage. In general the cell cycle is classified into five phases: the G_0_, G_1_, S, G_2_ and the M phases. The G_0_ is when the cell has left the cycle, G_1_ is the growth phase, S is the DNA replication phase, G_2_ is the gap between DNA synthesis and cell division, and M is the phase where cell division occurs. We investigated whether CPt associated with P4C6 vesicles (CPt-P4C6) is able to retain the same cell cycle modulation effect as CPt by using PI staining. Whereas flow cytometric analysis of blank P4C6 vesicles–treated SKOV-3 cells showed no cell cycle effects after 12 h of treatment, an increased percentage of cells in the S and G2/M phases and a corresponding decrease in G0/G1 cells were observed following 12 h of treatment with CPt and CPt-P4C6. (Fig. 4F) Compared to CPt, the CPt-P4C6 host-guest complex led to an increase of cells in the S phase from 21.2 ± 4.9% to 40.5 ± 9.9% and a decrease of cells in the G0/G1 phase from 42.8 ± 5.8% to 23.5 ± 4.6%. These data suggest that the increased effect of CPt-P4C6 on SKOV-3 cell proliferation resulted from an increase of the fraction of cells in the S phase, which is consistent with the mechanism of action of CPt. This result combined with previous findings shows the P4C6-carboplatin vesicles to be an effective platform for facilitating the delivery of carboplatin into the cell nucleus.

### Summary

We have established that the sparingly water soluble amphiphilic phosphonated calixarene lipid mimic P4C6 self-assembles into stable vesicles above 100 nm in diameter within a polar solvent. The vesicles can bind the water-soluble anti-cancer drug carboplatin within the cavity of the calixarenes which are restricted to be in the cone conformation, with 75% binding efficiency using a vortex fluidic device (VFD) under continuous flow mode of processing, which further highlights the versatility of this processing technology. The binding of the carboplatin in P4C6 was investigated using ^1^H NMR, FTIR, ESI-MS, EFTEM, TEM, SEM and AFM, establishing a 1:1 P4C6 to carboplatin complex which represents a high loading efficiency of the drug. The host-guest complex has high stability in pH 7.4 biosample media, with flow cytometric analysis establishing that the carboplatin-P4C6 host-guest vesicles result in a 4.5 fold increase in efficacy at 1 μM and a 10 fold lower IC_50_ relative to carboplatin in the absence of the P4C6. When treated with CPt-P4C6, more cells were stopped in the S phase implying P4C6 vesicles are a highly efficient delivery system for carboplatin.

The calixarene lipid mimic vesicles offer a number of promising features for anticancer drug delivery applications, including (1) they can be tagged with a targeting agent, a fluorescent molecule or wrapped in a polymer without reducing the vesicle stability^3^ (2) they can transport polar agents like carboplatin^19^ in their interior space and non-polar drugs like curcumin^4^, by intercalation or encapsulation, (3) the phosphonated calixarene is non-toxic^4^ and a potent anti-oxidant^5^, (4) their diameter is an optimal size for targeting cancerous tissue^7^, (5) they are relatively stable at normal tissue pH but rapidly release the drug molecule at typical cancerous tissue close to pH 6, and (6) that they increase the efficiency of carboplatin on ovarian cancer cells 4.5 fold.

After injection into the body, the vesicles are prone to elimination from systemic circulation by the reticuloendothelial system. This can be overcome by linking the vesicles to folic acid through a PEG moiety, noting that such PEGylation of vesicles can prolong residence time in the body, decrease degradation by metabolic enzymes and reduce or eliminate protein immunogenicity.

## Methods

### Blank P4C6 Vesicles Synthesis

Blank P4C6 vesicles were synthesised by a thin film method of preparation. P4C6 (17 g, 15 mmol) was weighed and dissolved in chloroform (50 ml) with ultrasonication for 5 minutes (FXP8D ultrasonic cleaner, Unisonics Australia, Brookvale, NSW, AUS). The solution was attached to a Buchi Rotavapor® R-215 (Flawil, Switzerland) to allow the evaporation of the organic solvent at 55° over 150 minutes. The resultant thin film was rehydrated in MilliQ water to give the desired P4C6 concentration (10 mM). Rehydration was performed by ultrasonication for 5 minutes followed by probe sonication (VCX130 ultrasonic processor, Sonics and Materials Inc. Newton, CT, USA) at 80% amplitude for 10 minutes to allow for the assembly of the nanoparticles. The final vesicles dispersion was filtered through a 0.45 μm PES Membrane filter (Millipore Corporation, Darmstadt, Germany).

### Carboplatin-P4C6 Binding

#### Vortex Fluidic Device (VFD)

Carboplatin (371 mg, 1 mmol) dissolved in water (100 ml) to give a 10 mM solution. This solution was delivered in one jet feed and a solution of the sized vesicles (10 mM P4C6) was delivered the second jet feed and passes through the VFD. The combined solution was passed through the VFD two more times. The rotational speed of the VFD was set at 8000 rpm, the flow rate at 0.6 mL/min, and the inclination angle of the tube at 45°. The supernatant vesicle dispersion was centrifuged (Heraeus Megafuge 40R Refrigerated Centrifuge, Thermo Fisher Scientific Inc., MA, USA) at 25,000 g for 30 min to precipitate the P4C6-carboplatin host-guest vesicles. The resultant pellet was washed twice with PBS (pH 7.4). The pellet was then suspended in PBS (pH 7.4) to make the final stock concentration of 10 mM CPt-P4C6 vesicles for further use.

#### Sonication

Blank P4C6 vesicles (5 mL, 10 mM) and carboplatin (5 mL, 10 mM) were mixed and then sonicated for 30 minutes (Elma Hans Schmidbauer GmbH & Co., Singen, Germany) at 80% magnitude. The supernatant dispersion was centrifuged (Heraeus Megafuge 40R Refrigerated Centrifuge, Thermo Fisher Scientific Inc., MA, USA) at 25,000 g for 30 min to precipitate the P4C6-carboplatin host-guest vesicles. The resultant pellet was washed twice with PBS (pH 7.4). The pellet was then suspended in PBS (10 ML, pH 7.4).

#### Drug Release

The 10 mM stock solution of VFD loaded carboplatin-P4C6 vesicles (2.5 ml) was injected into a dialysis bag (Spectrum Laboratories, Inc., Rancho Dominguez, CA, cut off molecular weight of 3500 Da) containing PBS (1000 ml) buffered to pH 5.5 or 7.4 and with 1% Tween 80 and 0.5% FBS, at 37.5 °C. At specific intervals the release medium around the dialysis bag was sampled (5 mL) for analysis by the established LC/TOF MS method mentioned below^17^.

#### LC/MS

Chromatography was performed using a WATERS Prominence (Waters MS Technologies, Manchester, UK) equipped with a 100 × 3.0 mm Symmetry C_18_ 3.5 μm column (Sunfire, Waters Corp, Milford, USA). Mass spectrometry was performed on a Micromass LCT Premier system (Waters MS Technologies, Manchester, UK) operating in positive ion mode. Gradient elution of a mobile phase comprising acetonitrile (A) and 1 mM aqueous sodium acetate (B) was delivered at a flow rate of 0.25 ml/min as follows: 0 – 3 min: 20% A; 3 – 6 min, linear increase of A from 20% to 80%; 6 – 9 min: 80% A; 9 to 12 min, linear decrease of A from 80% to 20%. Calibration standard solutions were prepared by appropriate dilutions of stock solutions of CPT (in water). The total analytical time was only 12 min. Detection and quantitation were performed by electrospray ionization (ESI) in the positive ionization mode with Selective Ion Monitoring (SIM) at *m*/*z* 310.0152. The calibration curves were linear over the concentration range of 10-4000 ng/ml, with the respective lower limit of quantification (LLOQ) at 10 ng/ml. The intra- and inter-day precision and accuracy of analysis of the quality control samples at low, medium, and high concentration levels were ≤13.6% relative standard deviation (RSD) and ≤14.6% relative errors (RE).

#### IC_50_

The SKOV-3 cells were seeded in 6-well plates (2 mL/well) with a concentration of 1 _×_ 10^6^ cells/mL in McCoy’s 5 A (modified) media (Life Technologies, San Diego, CA) and were incubated for 24 h in a humidified atmosphere containing 5% CO_2_ at 37 °C. The media in the wells was then replaced with prepared growth media (2 mL) which also contained P4C6, carboplatin or P4C6-carboplatin complex at various concentrations. The treated cells were incubated for a further 24 h, after which the sample-containing media was removed by aspiration. Cells in the media were collected by centrifugation on 275 *g* for 3 min. The remaining cell layers were washed with PBS buffer (2 × 1 mL). The total cells from cultures were differentially stained with Dead Cell Apoptosis Kit (Life Technologies, San Diego, CA) which contained two fluorescent dyes (Annexin V FITC and PI), allowing assessment of the viable, dead, and apoptotic cells. The number of viable cells was counted by a BD FACSCalibur flow cytometer (Becton, Dickinson and Company, Franklin Lake, NJ, USA) and was plotted against the total P4C6 or carboplatin concentration in logarithmic scale. The data reported represent an average of three measurements from different batches. The dose-response curves shown in Fig. 4 were obtained by sigmoidal logistic fitting using Origin 8.5 (OriginLab Corporation, Northampton, MA,USA) and the half-maximal inhibitory concentration (IC_50_) values were determined on the basis of the fitted data.

#### Cell Cycle Analysis

Samples-induced effects on SKOV-3 cells cycle were analysed using a FACS Calibur Flow Cytometer (Becton, Dickinson and Company, Franklin Lake, NJ, USA) with the BD Bioscience CellQuest Pro software (Becton, Dickinson and Company, Franklin Lake, NJ, USA). After exposure three different samples for 12 h SKOV-3 cells were harvested for counting of cell numbers.

Wash cells by centrifugation (300 g, 5 min, 4 °C) in PBS. Resuspend 2 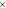 10^6^ cells in 1 ml ice cold PBS. Vortex gently and slowly adding the cell suspension dropwise to an equal volume of cold absolute ethanol. Store at 4 °C to –40 °C for overnight. Centrifuge cells at 300 g for 10 min at 4 °C. Resuspend pellet in 3 ml cold PBS and transfer to Falcon^TM^12 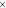 75 mm (FAL352003) polystyrene tubes for staining.

Wash cells at least once with cold PBS. Resuspend cells in 300 - 500 μl PI/Triton X-100 (Sigma-Aldrich Corporation, St. Louis, MO, USA) staining solution. Incubate 37 °C for 15 minutes. Transfer tubes to ice or store at 4 °C protected from light. Acquire data on flow cytometer and FlowJo software (FlowJo. LLC, Ashland, Oregon, USA) is used to fit the data to various cell cycle models.

## Additional Information

**How to cite this article**: Mo, J. *et al.* Shear induced carboplatin binding within the cavity of a phospholipid mimic for increased anticancer efficacy. *Sci. Rep.* **5**, 10414; doi: 10.1038/srep10414 (2015).

## Supplementary Material

Supporting Information
