# Supplementary material for: Shear induced carboplatin binding within the cavity of a phospholipid mimic for increased anticancer efficacy
Source: Sci Rep. 2015 May 22;5:10414. doi: 10.1038/srep10414 (PMC5386247; doi:10.1038/srep10414)
Supplement: Supporting Information [file srep10414-s1.doc]

Shear induced carboplatin binding within the cavity of a phospholipid mimic for increased anticancer efficacy

Jingxin Mo, Paul K. Eggers, Xianjue Chen, Thomas Becker, Muhammad Rizwan Hussain Ahamed, Lee Yong Lim and Colin L. Raston


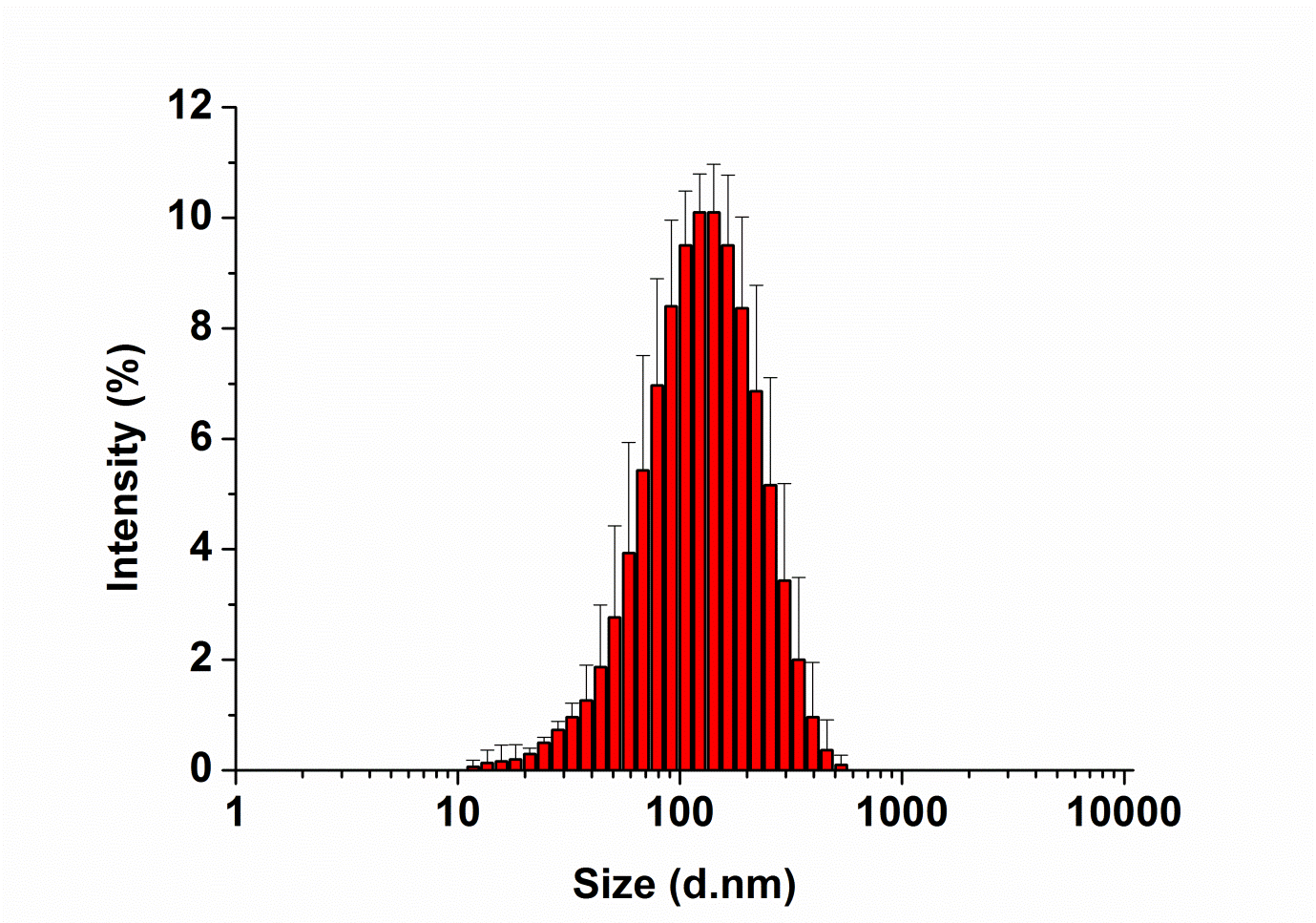


**Figure S1.** DLS of P4C6 vesicle devoid of carboplatin. The Z-average size is 107 ± 19 nm.


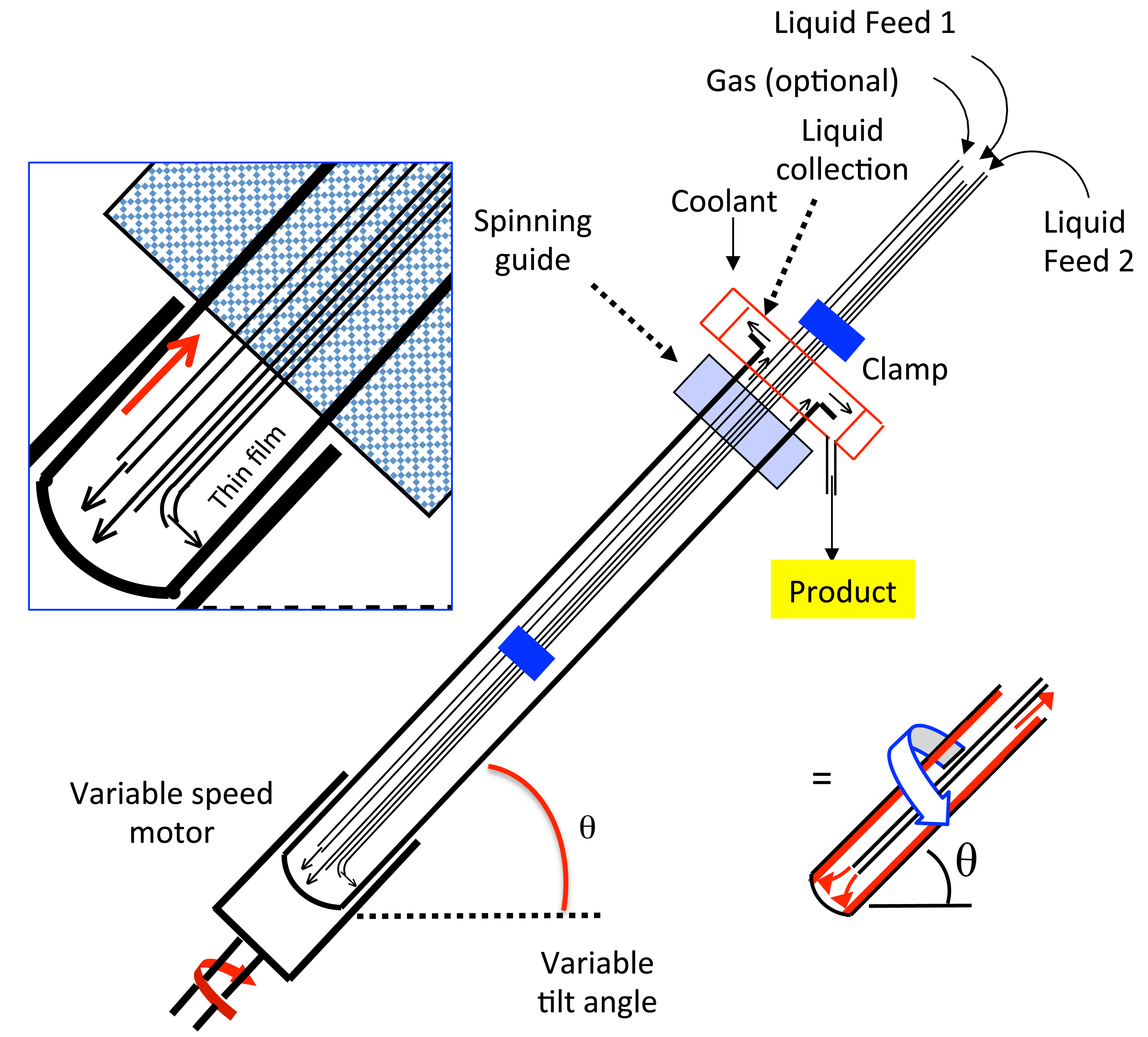


**Figure S2.** The Vortex Fluidic device (VFD)


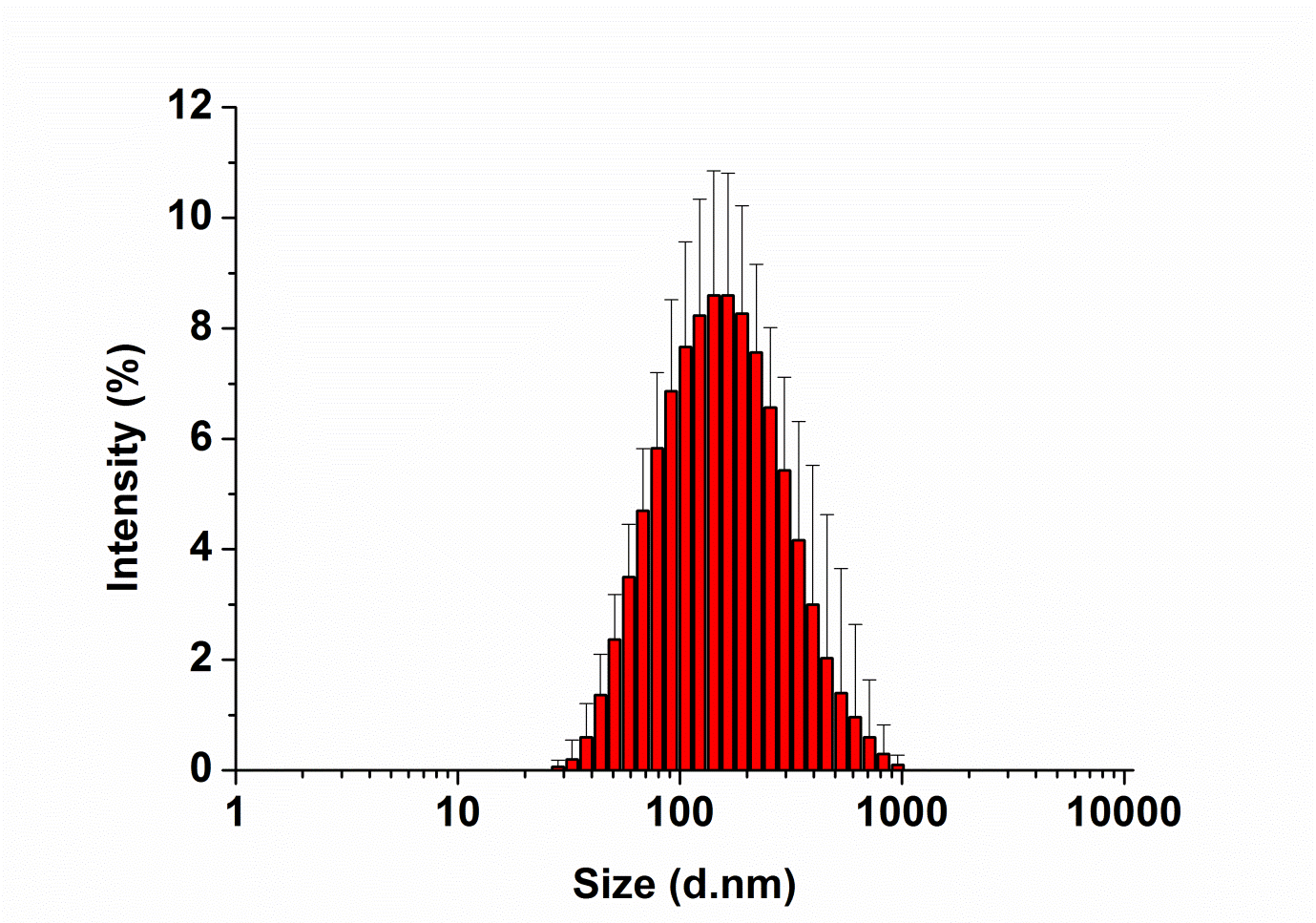


**Figure S3.** DLS of P4C6 vesicles loaded with carboplatin after VFD processing. The Z-average size is 134±25 nm.

General P4C6 micelle formation using the base-acid procedure

P4C6 was added to a solution at pH 10 made up from MilliQ water and 1 M NaOH. The mixture was readjusted to pH 10 with 1 M NaOH where applicable, stirred until P4C6 was completely dissolved, and then the pH was adjusted to pH 7.0 with 1 M HCl. Particles sizes were measured using DLS.


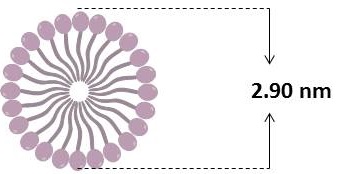

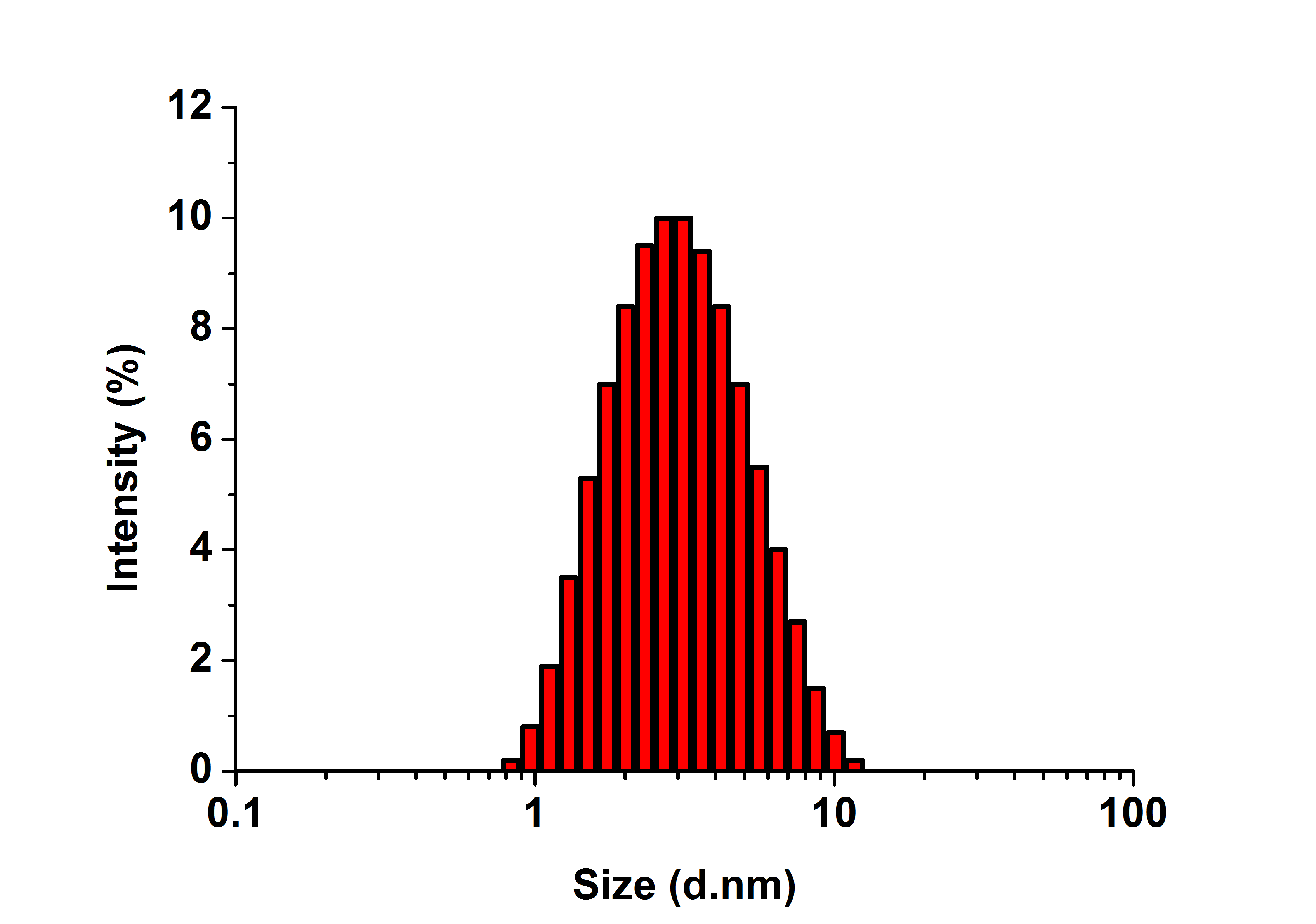


**Figure S4.** DLS of P4C6 micelles. The Z-average size is 2.90 nm.


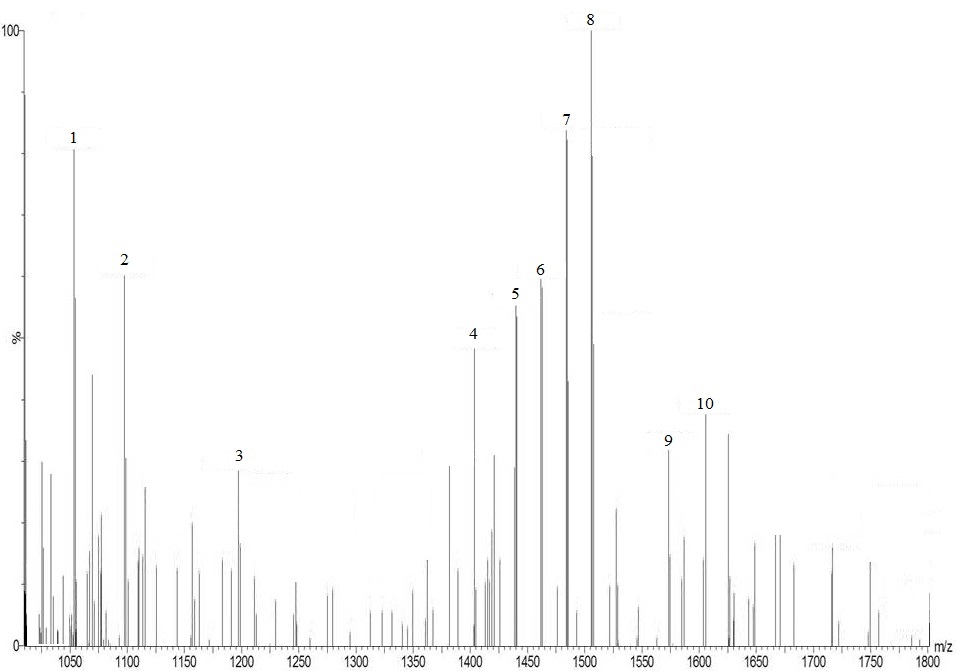


**Figure S5.** The electrospray ionisation mass spectra (positive mode) for the carboplatin-laden vesicle. Peaks are assigned as: 1. 1053.6107 m/z, [P4C6-PO_3_H_2_^-^+2H]^+^; 2. 1097.6483 m/z, [P4C6-2H_2_O+3H]^+^; 3. 1197.2875m/z, [P4C6+3Na^+^-8H]^+^; 4. 1403.7819 m/z, [P4C6+carboplatin-C_6_H_13_^-^-NH_3_-2H]^+^; 5. 1439.7605 m/z, [P4C6+carboplatin+Na^+^-C_6_H_13_^-^-6H]^+^; 6. 1461.7697 m/z, [P4C6+carboplatin-CO_2_-2H]^+^; 7. 1483.7225 m/z, [P4C6+carboplatin+Na^+^-CO_2_-3H]^+^; 8. 1508.7216 m/z, [P4C6+carboplatin+H]^+^; 9. 1573.5232 m/z, [P4C6+carboplatin+3Na^+^-2H]^+^; 10. 1605.6901 m/z, [P4C6+carboplatin+5Na^+^-NH_3_]^+^.

Electrospray Mass Spectrometry (ESI-MS) affording peaks corresponding to the empty container molecular, the container molecular with one carboplatin molecule, and the container molecular with a breakdown product of carboplatin, as noted in previous MS studies.[^1^](#_ENREF_1)


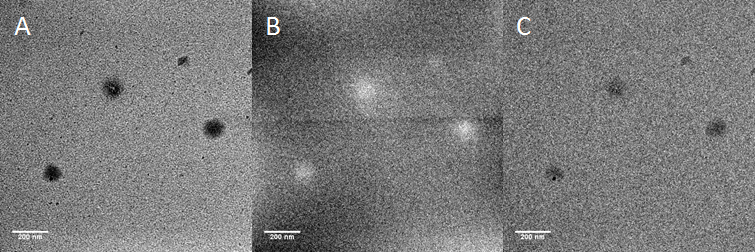


**Figure S6 Elemental mapping of blank P4C6 vesicles with energy-filtered transmission electron microscopy (EFTEM) for (A) unfiltered, (B) carbon and (C) platinum.**


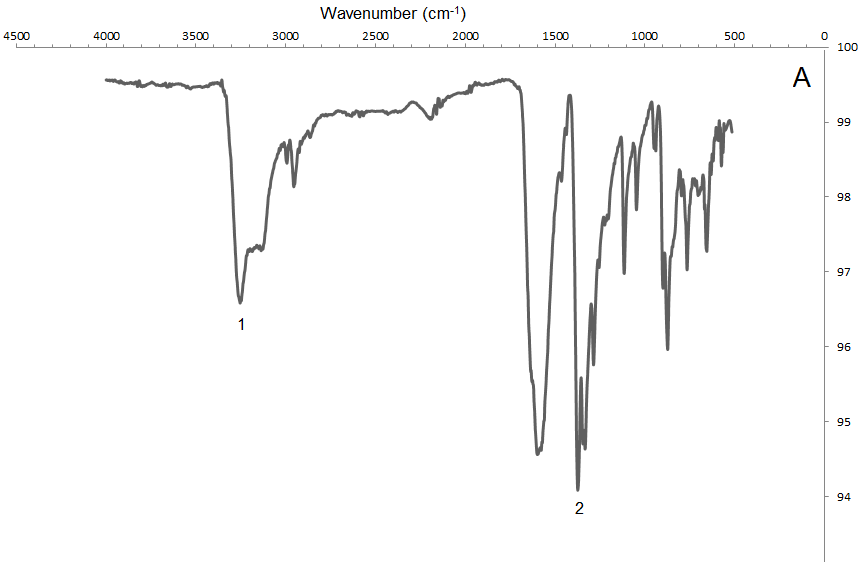


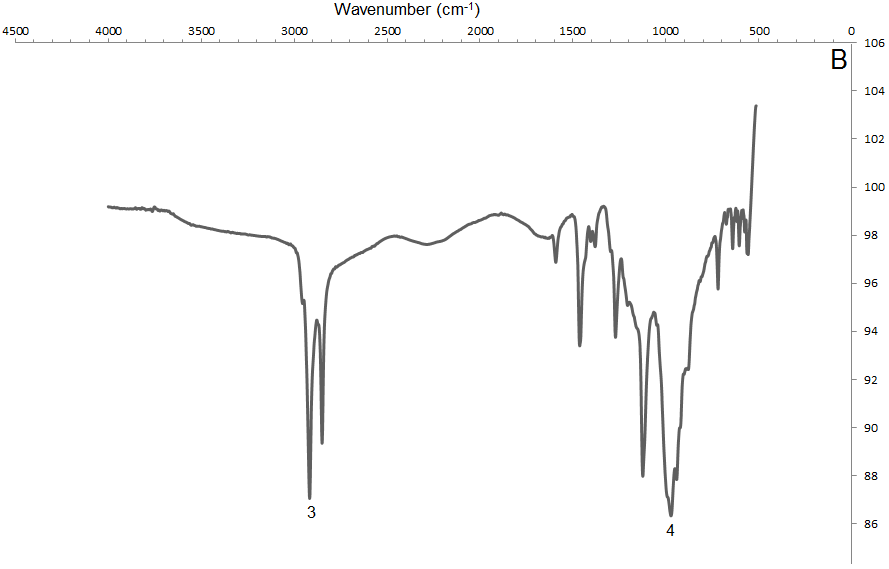


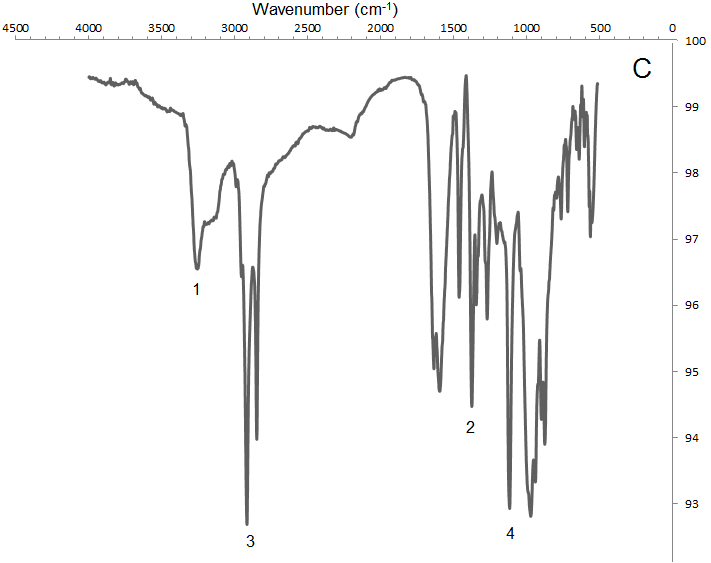


**Figure S7.** The FTIR spectrum of solid (A) carboplatin, (B) P4C6 and (C) vesicles based on P4C6 laden with carboplatin, in the range of 500–4000 cm^-1^.

FTIR spectra of carboplatin, P4C6 and the complex with carboplatin are given in Fig. S7. Characteristic bands observed are assigned as following: 1(3251-3339 cm^-1^) are νa(NH_3_); 2(1138-1341 cm^-1^) are δs(NH_3_), twist CH_2_, o-ph ν(C–O), in-ph ν(C–O); 3(2798-2988 cm^-1^) are νs (P=O), νa (P=O); 4(1059-1096 cm^-1^) are ν(C–O), Twist CH_2_. In particular, the shift of carboplatin ν_a_(NH_3_) from 3253s cm^-1^ (Figure S7A) to 3339s cm^-1^ (Figure S7C), indicating NH⋯O hydrogen bond formation.

*
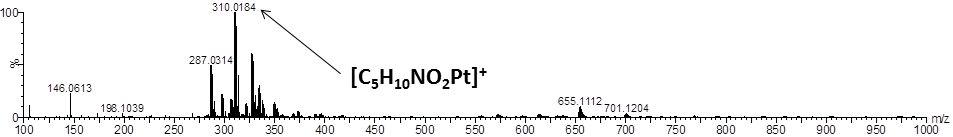
*

**Figure S8.** The generation of ([C_5_H_10_NO_2_Pt]^+^) mass spectra of carboplatin.


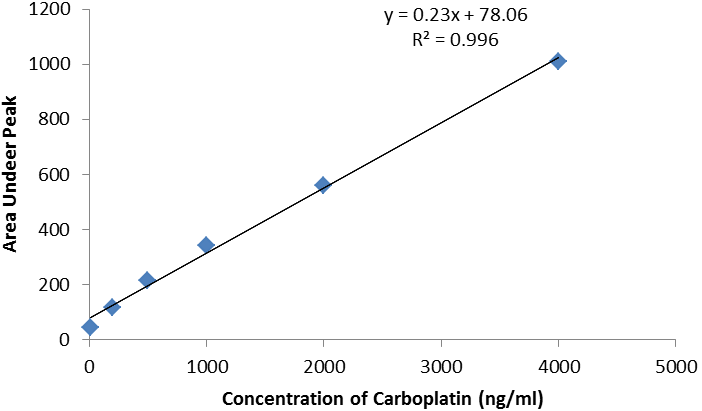


**Figure S9.** The standard curve for carboplatin. Calibration standards containing carboplatin in the concentrations (ng/mL) of 10, 200, 500, 1000, 2000, and 4000 were prepared before injection into LC/TOF MS for analysis. Calibration curves of carboplatin exhibited excellent linearity over the concentration ranges of 10-4000 ng/mL.

**Preparation of Calixarenes**

**Preparation of 5,11,17,23-Tetra-formyl-25,26,27,28-tetra-hexyloxy-calix[4]arene**

Under inert gas atmosphere a mixture of dry 25,26,27,28-tetra-hexyloxy-calix[4]arene (11.0 g, 14.5 mmol) and hexamethylenetetramine (78 g, 560 mmol ) in CF_3_COOH (400 mL) was stirred for 96 h under reflux. The mixture was cooled to room temperature and then poured into a stirring solution of 2 M HCl (500 mL) and CH_2_Cl_2_ (500 mL), and vigorously stirred for 1 h. The mixture was extracted with CH_2_Cl_2_ (2 x 300 mL) and the combined organic layers were washed with saturated aqueous Na_2_CO_3_ (2 x 300 mL) and brine (2 x 300 mL), dried over NaSO_4_ and the solvent was then removed under reduced pressure. The raw product was further purified by column chromatography (silica gel 60; hexane:ethyl acetate, 7:3) to give a white solid (9.0 g, 71 %).

^1^H NMR (CDCl_3_, 500 MHz) δ: 0.92 (t, ^3^*J*=6.95 Hz, 12H), 1.36 (m, 24H), 1.88 (m, 8H), 3.34, 4.49 (2d, *^2^J*=13.9 Hz, 2 x 4H) 3.96 (t, ^3^*J*=7.51 Hz, 8H), 7.15 (s, 8H), 9.58 (s, 4H).

**Preparation of 5,11,17,23-Tetra-methylhydroxy-25,26,27,28-tetra-hexyloxy-calix[4]arene**

Under an inert gas atmosphere ethanol (200 mL) was added to a stirring solution of 5,11,17,23-tetra-formyl-25,26,27,28-tetra-hexyloxy-calix[4]arene (9.0 g, 10.3 mmol) in THF (80 mL). NaBH_4_ (4.67 g, 123 mmol) was then added and the mixture stirred for 18 h at room temperature. The mixture was then concentrated under vacuum and the resulting solid dissolved in CH_2_Cl_2_ (200 mL). 2 M HCl (200 mL) was added slowly and the solution was stirred for 1 h. The reaction mixture was then extracted with CH_2_Cl_2_. The combined organic fractions were washed with 2 M HCl (3 x 200 mL) and saturated aqueous NaHCO_3_ (1 x 200 mL) and dried over Na_2_SO_4_ .The solvent was removed *in vacuo* to obtain a white solid (8.3 g, 91 %).

^1^H NMR (CDCl_3_, 500 MHz) δ: 0.91 (t, ^3^*J*=6.91 Hz, 12H), 1.36 (m, 24H), 1.91 (m, 8H), 3.13, 4.42 (2d, *^2^J*=13.1 Hz, 2 x 4H), 3.87 (t, ^3^*J*=7.78 Hz, 8H), 4.31 (s, 8H), 6.69 (s, 8H).

**Preparation of 5,11,17,23-Tetra-diethylphosphonomethyl-25,26,27,28-tetra-hexyloxy-calix[4]arene**

Under an inert gas atmosphere, thionylchloride (30 mL) was added to 5,11,17,23-tetra-methylhydroxy-25,26,27,28-tetra-hexyloxy-calix[4]arene (3.0 g, 3.4 mmol) and then stirred at room temperature for 18 h. The mixture was concentrated under vacuum and the resulting solid was dissolved in CH_2_Cl_2_ (200 mL), washed with saturated aqueous NaHCO_3_ (1 x 100 mL) and dried over Na_2_SO_4_ .The solvent was removed under reduced pressure to obtain an off-white solid.

Dry 5,11,17,23-Tetra-methylchloride-25,26,27,28-tetra-hexyloxy-calix[4]arene was added with stirring to triethylphosphite (150 mL) and the mixture was then refluxed for 16 h. The solution was cooled to room temperature and the triethylphosphite removed under reduced pressure. The product was purified by flash column chromatography (silica gel 60; chloroform:methanol, 10:1) to give a clear glass (3.61 g, 78 %).

^1^H NMR (CDCl_3_, 500 MHz) δ: 0.89 (t, ^3^*J*=6.98 Hz, 12H), 1.20 (t, ^3^*J*=7.09 Hz, 24H), 1.32 (m, 24H), 1.82 (m, 8H), 2.76 (d, *^2^J*=21.2 Hz, 8H), 3.05, 4.33 (2d, *^2^J*=13.4 Hz, 2 x 4H), 3.80 (t, ^3^*J*=7.55 Hz, 8H), 3.93 (m, 16H), 6.49 (s, 8H).

^13^C NMR (CDCl_3_, 125.8 MHz) δ: 14.0 (CH_3_), 16.3 (d, CH_3_, 6.2 Hz), 22.7 (CH_2_), 25.9 (CH_2_), 30.1 (CH_2_), 30.9 (CH_2_), 32.0 (CH_2_), 33.0 (d, CH_2_, 138 Hz), 61.9 (d, CH_2_, 6.6 Hz), 75.1 (CH_2_), 124.0 (d, C, 9.1 Hz), 129.4 (d, CH, 6.4 Hz), 135.0 (d, C, 3.1 Hz), 155.8 (d, C, 4.1 Hz).

**Preparation of 5,11,17,23-Tetra-phosphonomethyl-25,26,27,28-tetra-hexyloxy-calix[4]arene**

Under an inert gas atmosphere, bromotrimethylsilane (3 mL) was added to dry 5,11,17,23-tetra-diethylphosphonomethyl-25,26,27,28-tetra-hexyloxy-calix[4]arene (1.0 g, 0.73 mmol) in acetonitrile (40 mL) and was refluxed for 15 h. The bromotrimethylsilane was removed under reduced pressure and acetonitrile (30 mL) was added. Water (1mL) was added and stirred slowly for 2 h. The resultant white solid that formed was collected *via* vacuum filtration (0.79 g, 95 %).

^1^H NMR (MeOD/CDCl_3_, 500 MHz) δ: 0.95 (t, ^3^*J*=7.17 Hz, 12H), 1.41 (m, 24H), 1.95 (m, 8H), 2.84 (d, *^2^J*=21.0 Hz, 8H), 3.10, 4.42 (2d, *^2^J*=12.7 Hz, 2 x 4H), 3.86 (t, ^3^*J*=7.16 Hz, 8H), 6.67 (s, 8H).

^13^C NMR (MeOD/CDCl_3_, 150.9 MHz) δ: 13.3 (CH_3_), 22.6 (CH_2_), 25.9 (CH_2_), 30.2 (CH_2_), 30.4 (CH_2_), 32.0 (CH_2_), 33.6 (d, CH_2_, 136 Hz), 75.0 (CH_2_), 125.6 (d, C, 9.0 Hz), 129.6 (d, CH, 7.0 Hz), 134.6 (d, C, 2.6 Hz), 155.1 (d, C, 3.7 Hz).

TOF MS ES­­^-^ (*m/z*), Expected for C_56_H_83_O_16_P_4_^-^ [M-H]^-^: 1135.4632, Found: 1135.4625.

**REFERENCE:**

1. Mo JX, Eggers PK, Raston CL, Lim LY. Development and validation of a LC/TOF MS method for the determination of carboplatin and paclitaxel in nanovesicles. *Analytical and bioanalytical chemistry* 2014, **406**(11)**:** 2659-2667.
